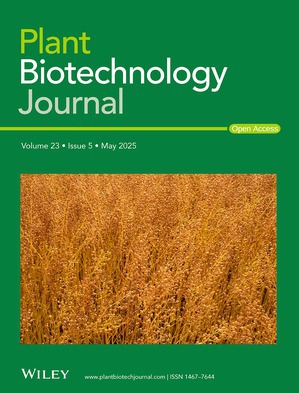# Issue Information

**DOI:** 10.1111/pbi.14391

**Published:** 2025-04-24

## Abstract

Front cover image:

A close up of a field of camelina at maturation. The ‘seed pods’ are spherical. The crop at maturation looks like flax and that is why camelina is also called ‘false flax’. The proper botanical term for the seed pods is ‘silicles’ (length/width ratio is smaller than in standard siliques found in Brassicaceae). These siliques contain 10 to 20 small orange/brown seeds.

Photograph by E.N. van Loo, Wageningen University and Research.

Cover illustration refers to the article published in this issue (Belle et al., pp. 1399–1412).